# The Multiple Faces of Nodular Trichoblastoma: Review of the Literature with Case Presentation

**DOI:** 10.3390/dermatopathology8030032

**Published:** 2021-07-05

**Authors:** Gerardo Cazzato, Antonietta Cimmino, Anna Colagrande, Francesca Arezzo, Lucia Lospalluti, Sara Sablone, Teresa Lettini, Leonardo Resta, Giuseppe Ingravallo

**Affiliations:** 1 Section of Pathology, Department of Organ and Emergency Transplantation (DETO), University of Bari “Aldo Moro”, 70124 Bari, Italy; micasucci@inwind.it (A.C.); anna.colagrande@gmail.com (A.C.); lettinit@yahoo.com (T.L.); leonardo.resta@uniba.it (L.R.); 2 Section of Gynecology and Obstetrics, Department of Biomedical Sciences and Oncology (DIMO), University of Bari “Aldo Moro”, 70124 Bari, Italy; francesca.arezzo@uniba.it; 3 Section of Dermatology, Department of Biomedical Sciences and Oncology (DIMO), University of Bari “Aldo Moro”, 70124 Bari, Italy; l.lospalluti@gmail.com; 4 Section of Legal Medicine, Department of Interdisciplinary Medicine, Policlinico di Bari Hospital, University of Bari “Aldo Moro”, 70124 Bari, Italy; sarasabloneml@gmail.com

**Keywords:** trichoblastoma, skin, adnexal tumors, dermatopathology

## Abstract

Trichoblastoma (TB) is a rare biphasic benign adnexal neoplasm originating from follicular germ cells but clinically, it can simulate basal cell carcinoma (BCC), making the diagnosis more difficult. There are several variants of Trichoblastoma and a good knowledge of these is essential for correct diagnosis and management. We report two new cases observed in the last year at our Pathological Anatomy Operative Unit, and conduct a careful review of the literature, from the first description of this lesion by Headington in 1970 to the most recent classifications.

## 1. Introduction

Trichoblastoma (TB) is a rare biphasic benign adnexal neoplasm originating from follicular germ cells which is classified in the latest WHO “Skin Tumours” classification (2018) among lesions with a favorable clinical outcome [1,2], although rare cases of trichoblastic carcinoma as an evolution of TB have been described [3]. Clinically, this lesion can simulate basal cell carcinoma (BCC), making the diagnosis more difficult. Furthermore, there are several variants of Trichoblastoma and a good knowledge of these is essential for correct diagnosis and management. Here, we report two new cases observed in the last year at our Pathological Anatomy Operative Unit, and conduct a careful review of the literature, from the first description of this lesion by Headington in 1970 to the most recent classifications.

## 2. Materials and Methods

We conducted a literature review using Pubmed and Web of Science (WOS) search engines, entering the words “trichoblastoma” OR “cutaneous trichoblastoma” OR “trichoblastic carcinoma” and/or “adnexal neoplasm” OR “cutaneous adnexal neoplasm” to retrieve descriptions referring to this entity. Together with the review, we report two clinical cases diagnosed at our unit in the last year after complete histopathological analysis.

### 2.1. Case 1

**Figure 1:**
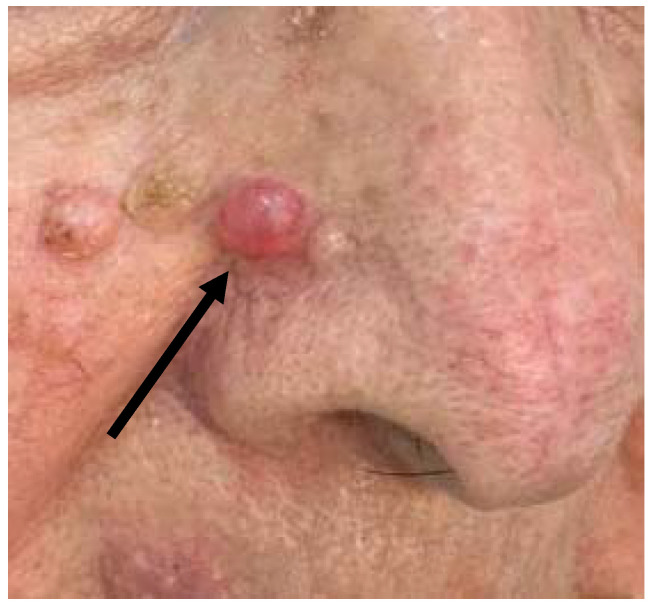
Clinical features of the “Case 1” lesion.

The first case was a 73-year-old woman who complained of the persistence of a skin lesion at the level of the right nasolabial groove (Figure 1), that had been present for several years. The patient reported frequent traumas and subsequent bleeding, that prompted her to refer to a dermatologist. At the clinical level, the lesion appeared as a well circumscribed symmetrical pinkish mass with beveled edges, measuring 1.2 cm in diameter. Clinical diagnosis of basal cell carcinoma or, secondarily, of a sebaceous cyst was made. The lesion was excised and sent to the Pathology Laboratory.

### 2.2. Case 2

**Figure 2:**
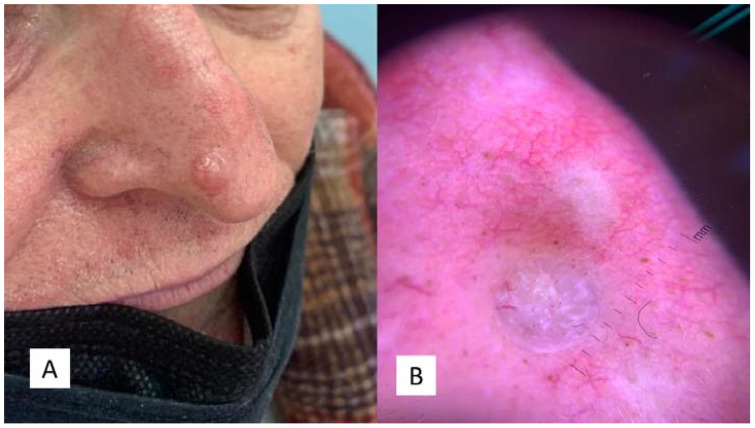
(**A**) Clinical features of the “Case 2” lesion, well circumscribed, symmetrical, smooth bordered, skin-colored pinkish or brown, at the level of the passage from the tip to the right nasal wing. (**B**) Dermoscopic features of TB with arborizing vessels and teangiectasias.

The second case was a 67-year-old man who had suffered for an unspecified number of years from a cyst-like lesion of the nose. He had no history of trauma and/or bleeding, but for aesthetic reasons he had decided to refer to the dermatologist. Clinically, the mass was solitary, well circumscribed, nodular, measuring about 1.0 cm in diameter (Figure 2A,B). Surgical removal was carried out and the specimen was sent to the Pathology Laboratory.

Both specimens were subjected to sampling, processing, paraffin embedding, microtome cutting and Hematoxylin/Eosin (H&E) staining.

## 3. Results

**Figure 3:**
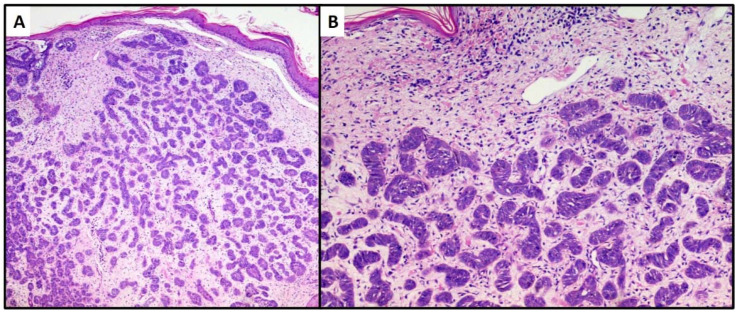
Proliferation of numerous small and irregular nests of basaloid cells with no obvious connection to the epidermis (hematoxylin and eosin, original magnification, (**A**) 40× (**B**) 100×).

The first lesion consisted of a proliferation of basaloid cells (high N/C ratio) localized in the middle and deep dermis, with no obvious connection to the epidermis. This lesion consisted of small, irregular nests of basaloid cells (variant with small nodules of TB), surrounded by a stroma of fibrocytes and various pilar differentiation foci (Figure 3A). The neoplasm did not reach the subcutis. In view of the absence of a clear peripheral “palisade” and mild mitoses (Figure 3B), the diagnosis of the Trichoblastoma variant “with small nodules” was made. The margins were disease-free, and no disease recurrence had occurred at 1 year of follow-up.

**Figure 4:**
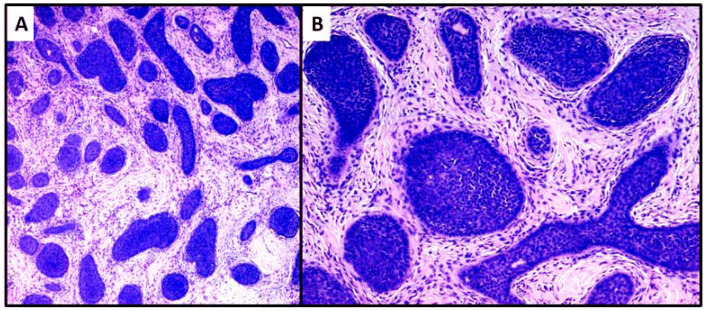
Proliferation of large nests of basaloid cells without connection to the epidermis (hematoxylin and eosin, original magnification, (**A**) 40× (**B**) 100×).

The second case was characterized by a population of basaloid cells aggregated in large nodules, with some evidence of a “peripheral palisade” (Figure 4A,B). There was no connection with the overlying epidermis, but there was a marked tendency to deep infiltration down to the muscular plane. The mitotic index, in this second case, was higher and owing to the greater aggressiveness of the lesion, extended surgical resection margins and careful follow-up were decided upon. After radical surgery with negative margins for neoplasia, at 18 months of follow-up, no recurrence of the disease was observed.

## 4. Discussion

Trichoblastoma is a rare benign tumor of the follicular germ cells, in which follicle development may be partly or completely replicated [4]. It was first described by Headington as a differentiated follicular neoplasm [5]. In the following years, different nomenclatures were adopted, including “trichogenic tumor” [6] or “trichogenic trichoblastoma” [7]. In a 1995 review, Schirren et al. [8] reviewed the knowledge of this entity and since then, the term Trichoblastoma has been exclusively used. Usually, a Trichoblastoma measures more than 1 cm in diameter and may involve the deep dermis and/or subcutis [9]. Our two new cases were also 1 cm or more, and both showed a tendency to infiltrate the deep dermis, and sometimes reach the subcutis. The parts of the body most affected are the head and neck districts [2], although other localizations have also been reported, such as the vulva [10]. In addition, various cases of lesions joining TB with other cutaneous neoplasms have been reported: for example, with inverted follicular keratosis [11], or with syringocystoadenoma papilliferum in a common sebaceous nevus [12], or again with a dilated pore of Winer [13]. There has been much debate about the importance of identifying dermoscopic criteria that could allow a correct differential diagnosis between TB and BCC, but a recent 2020 review [14] clarified that, despite the efforts of Ghigliotti et al. [15] in demonstrating significantly more blue–gray ovoid nests and globules in BCC compared to TB, these features may also be present in TB, so a confirmatory histopathological diagnosis is still necessary today. Many, perhaps most, dermatopathologists regard trichoepithelioma (TE) as a variant of trichoblastoma (TB) with smaller aggregations of cells; indeed, conventional and desmoplastic trichoepithelioma have also been referred to as cribriform and columnar trichoblastoma, respectively [2,6]. Requena L. et al., for example, consider trichoepithelioma to be a superficial variant of trichoblastoma, showing characteristics of a benign, symmetrical and well-defined tumor consisting mainly of germinative follicular cells embedded in an abundant fibrotic stroma [6].

In the latest WHO 2018 “Skin Tumors” classification [2], different variants of TB are recognized, making the differential diagnosis even more complex even for the dermatopathologist: nodular [16] (as in our two cases), retiform, cribriform [17], racemiform [18] and columnar patterns of TB. Furthermore, rare variants of TB include clear cell [18], pigmented (pigmented TB) [19,20] and adamantinoid lesions [21]. It is generally believed that another rare adamantine variant of TB is cutaneous lymphadenoma, a rare adnexal tumor with a prominent lymphocytic infiltrate in the tumor nests [22,23]. In addition to histopathology, various scientific works [24] have attempted to identify immunohistochemical markers that could reliably differentiate between TB and BCC, including CD34 positivity of the stroma fibrocytes in TB versus negative stroma fibrocytes in basal cell carcinoma [25,26,27]; different BCL-2 expression, that is diffusely positive in epithelial cells of BCC but only marks the peripheral layer of cells in TB [28,29]; finally, the number of CK20-positive Merkel cells, more abundant in TB than in BCC. Immunohistochemical expression of PHLDA1 (a marker of follicular stem cells) is positive in TB and completely negative in BCC [30]; conversely, markers such as CD10, follistatin and Bmi-1 have not yet been shown to offer a safe differential diagnosis [31]. Leonid Izikson et al. reported that expression of the androgen receptor could be more indicative of BCC than TB [32].

## 5. Conclusions

We report two new cases of nodular TB and a review of the main works in the literature describing this lesion. Even today, a certain diagnosis remains entirely histopathological, and although different immunohistochemistry markers have been studied, it is not yet routine practice to perform immunohistochemical analysis to differentiate TB from BCC. In short, cell morphology still plays a fundamental role.
